# The effectiveness of digital health interventions on anthropometric and healthy behavior in women with polycystic ovarian syndrome: a systematic review with meta-analysis

**DOI:** 10.3389/fendo.2026.1676915

**Published:** 2026-02-27

**Authors:** Hui-fang Zeng, Zhen Dou, Yi-Nuo Zhang, Chu-Chu Wang, Tian Xia, Jing Xu

**Affiliations:** 1 First Teaching Hospital of Tianjin University of Traditional Chinese Medicine, Tianjin, China; 2 National Clinical Research Center for Chinese Medicine Acupuncture and Moxibustion, Tianjin, China; 3 The People’s Hospital of Wuchuan, Zunyi, China

**Keywords:** anthropometric measures, digital health interventions, health behavior, polycystic ovary syndrome, systematic review

## Abstract

**Objective:**

To investigate the effects of digital health interventions (DHIs) for improving anthropometric and healthy behavior in women with polycystic ovarian syndrome (PCOS).

**Methods:**

Five databases were searched from their inception to April 2025 with no date restrictions. Standardized mean differences (SMDs) and mean differences (MDs) with 95% confidence intervals (CIs) were pooled using random−effects models. The certainty of the evidence was assessed using the GRADE approach.

**Results:**

Ten trials with 930 participants were included. In short−term (≤3 months), DHIs yielded significant reductions in BMI (MD –1.19; –1.84 to –0.55; I²=0%), waist circumference (MD –2.14; –3.11 to –1.17; I²=0%) and large improvements in total HPLP−II score (SMD 1.61; 1.20 to 2.01; I²=0%), physical activity (SMD 1.43; 1.04 to 1.83; I²=0%), health responsibility (SMD 1.02; 0.64 to 1.39; I²=0%), interpersonal relationships (SMD 0.96; 0.59 to 1.33; I²=0%), spiritual growth (SMD 1.25; 0.87 to 1.64; I²=0%) and stress management (SMD 1.17; 0.79 to 1.55; I²=0%); there was no significant change in body weight (SMD –0.04; –0.23 to 0.15; I²=0%) or HPLP−II nutrition (SMD 0.83; –0.11 to 1.78; I²=84%). In medium−term (3–6 months), DHIs continued to reduce BMI (MD –2.46; –3.04 to –1.88; I²=22%) and waist circumference (MD –4.65; –6.70 to –2.60; I²=0%), and yielded significant improvements in depressive symptoms (SMD –0.85; –1.17 to –0.53; I²=17%) and anxiety (SMD –0.95; –1.33 to –0.56; I²=42%).

**Conclusion:**

Digital health interventions confer significant short− and medium−term improvements in anthropometric and healthy behavior measures among women with PCOS.

**Systematic review registration:**

https://www.crd.york.ac.uk/prospero/, identifier CRD420251030598.

## Introduction

Polycystic ovary syndrome (PCOS) is recognized as the most prevalent endocrine disorder affecting women of reproductive age worldwide (1, 2), with recent meta-analytic evidence estimating its global prevalence at approximately 9.2% (95% CI, 6.8–12.5%) (3). Women with PCOS commonly present with a constellation of anthropometric abnormalities, including overweight, obesity, and central adiposity, which contribute to insulin resistance, dyslipidemia, and increased cardiometabolic risk (4, 5). Central adiposity in PCOS not only exacerbates metabolic dysfunction but also correlates with heightened psychological distress, including depression and anxiety (6), which further impairs health-related quality of life and self-management capacity (7).

Current first-line management of PCOS emphasizes lifestyle modification, including structured behavioral interventions targeting weight loss, dietary quality, and exercise. Several meta-analyses (8–10) have demonstrated that such interventions yield significant reductions in BMI and waist circumference, as well as improvements in depressive symptomatology and metabolic indices over short- to medium-term follow-up. Despite these benefits, traditional face-to-face programs are often hindered by barriers such as geographic inaccessibility, scheduling constraints, limited healthcare resources, and poor long-term adherence, resulting in variable effectiveness and sustainability of behavioral changes (11). Consequently, innovative delivery platforms are needed to enhance scalability, personalization, and continuous engagement with lifestyle interventions for women with PCOS.

Digital health interventions (DHIs) (12–14)—which encompass mobile applications, Internet of Things (IoT) devices, web-based platforms, wearable devices, and social media tools—have emerged as promising modalities for delivering scalable, cost-effective, and tailored lifestyle support for chronic conditions. In the context of PCOS, preliminary descriptive studies reveal high interest and utilization of digital technologies: up to 98% of women with PCOS search online for PCOS-specific information, and nearly one-fifth participate in web-based support groups, indicating a strong demand for digital self-management resources (11). Given the increasing global burden of PCOS, the high prevalence of obesity and unhealthy lifestyle behaviors among affected women, and the limitations of conventional behavioral interventions, there is an urgent need for a rigorous synthesis of the evidence on digital health strategies in this population.

To our knowledge, no comprehensive systematic review with meta-analysis has concurrently evaluated the impact of digital health interventions on anthropometric outcomes and health-promoting behaviors in women with PCOS. Accordingly, this study aims to systematically review and meta-analyze randomized controlled trials of digital health interventions in women with PCOS, focusing on changes in key anthropometric measures and health-promoting behaviors, as well as psychological outcomes. By quantifying the pooled effects, we seek to elucidate the current efficacy of digital interventions, identify critical research gaps, and provide recommendations for optimizing digital health strategies in this high-risk population.

## Methods

This systematic review was carried out following the methods of the Cochrane Handbook (15) for Systematic Reviews and reported by the Preferred Reporting Items for Systematic Review and Meta-Analyses (PRISMA) statement (16). The protocol has been registered on the International Prospective Register of Systematic Reviews (PROSPERO): CRD420251030598.

### Information sources and electronic searches

We performed a comprehensive literature search in April 2025 to capture all potentially eligible trials, irrespective of language or date of publication. Our search encompassed five major electronic databases: PubMed, EMBASE, the Cochrane Central Register of Controlled Trials (CENTRAL), Scopus, and Web of Science. The search strategy was developed and implemented by an independent medical librarian, with the complete algorithms provided in **Supplementary Material Table A**. To ensure comprehensive coverage, we augmented our database searches with backward citation tracking of all included articles and a review of reference lists from existing systematic reviews. For full texts that were not accessible through institutional subscriptions or interlibrary loans, we contacted corresponding authors via email to request manuscripts or clarify unpublished data. All citations were imported into EndNote for de-duplication, after which two independent reviewers screened titles, abstracts, and full texts against predefined eligibility criteria. Any discrepancies were resolved through consensus or, if necessary, by consulting a third reviewer.

### Study selection and data extraction

Two reviewers independently performed data extraction following the Cochrane Handbook for Systematic Reviews of Interventions (version 6.3) guidelines. For each eligible trial, we recorded study characteristics (author, year, country), participant demographics (age, sample size), digital health intervention details, comparator treatment details, and outcome measures. Eligible studies comprised randomized and quasi−randomized controlled trials evaluating the effectiveness of digital health interventions versus non-digital control for women with polycystic ovarian syndrome. Trials were included if they reported at least one of the anthropometric or healthy behavior measures. We divided outcomes into short term (less than or equal to three months), medium term (over three and up to six months), and long term (over six months). If sufficient studies are available, data from different periods will be extracted for meta-analyses. Animal trials and non-English studies were excluded. Non-English full-text articles were excluded because reliable translation resources were not available to ensure accurate data extraction and risk-of-bias assessment. Any disagreements were resolved by discussion or, if necessary, by consultation with a third reviewer.

### Risk of bias assessment

Individual studies were formally evaluated for risk of bias using the Cochrane risk of bias tool (version 2, ROB2) (17), assessing for the randomization process, intended interventions, missing outcome data, measurement of the outcome, and selection of the reported result. Each trial underwent assessment in these five bias domains, resulting in a summary risk-of-bias score for each domain and an overall classification (low risk, some concerns, or high risk of bias). Two authors assessed each of the included studies and each potential source of bias was graded as high, low, or unclear risk of bias. Two reviewers independently performed the assessment. Discrepancies were resolved by consensus or, if needed, through discussion with the research team.

### Data analysis

Pooled estimates of treatment effect for continuous outcomes were combined using either mean differences (MD) (if the same instrument was used) or standardized mean differences (SMD) and 95% CIs. We preferentially applied a random−effects model to account for between−study variability among trials judged to be clinically and methodologically comparable. Following Cochrane recommendations, if means or standard deviations were not directly reported, we estimated them from available *p* values, CIs, or standard errors. Where necessary, we contacted corresponding authors to retrieve missing summary data. Where published data were insufficient for inclusion in the meta−analysis, we requested unpublished subgroup or ancillary data directly from study investigators to minimize reporting bias. Following Cohen (18), the effect sizes were interpreted as: large (≥0.8), moderate (0.5-0.8), small (0.2-0.5), and trivial (<0.2). Statistical heterogeneity was evaluated for each pooled analysis using the I² statistic and categorized as follows: low (<25%), moderate (25–50%), substantial (50–75%), or considerable (>75%) (15). To test the robustness of our findings, we conducted sensitivity analyses by sequentially excluding individual trials and recalculating pooled estimates. Due to the small number of included trials in the meta-analyses (<10) (19), a formal assessment of publication bias via funnel plot asymmetry was not performed. All statistical computations were carried out in Review Manager (RevMan) version 5.4.1.

### Assessing the quality of the evidence

The certainty of evidence was assessed by using the GRADE (Grading of Recommendations Assessment, Development and Evaluation) approach (20). Two reviewers, both experienced in evidence synthesis, independently rated the quality of evidence across the five GRADE domains: risk of bias, inconsistency, indirectness, imprecision, and publication bias. For each meta-analysis, we began with a default “high” rating, reflecting the inclusion exclusively of randomized controlled trials. Ratings were then potentially downgraded by one or two levels for concerns in any domain. Discrepancies in domain judgments or overall certainty were resolved through consensus discussion; persistent discordance was adjudicated by a third reviewer.

## Results

### Study selection

**Figure 1:**
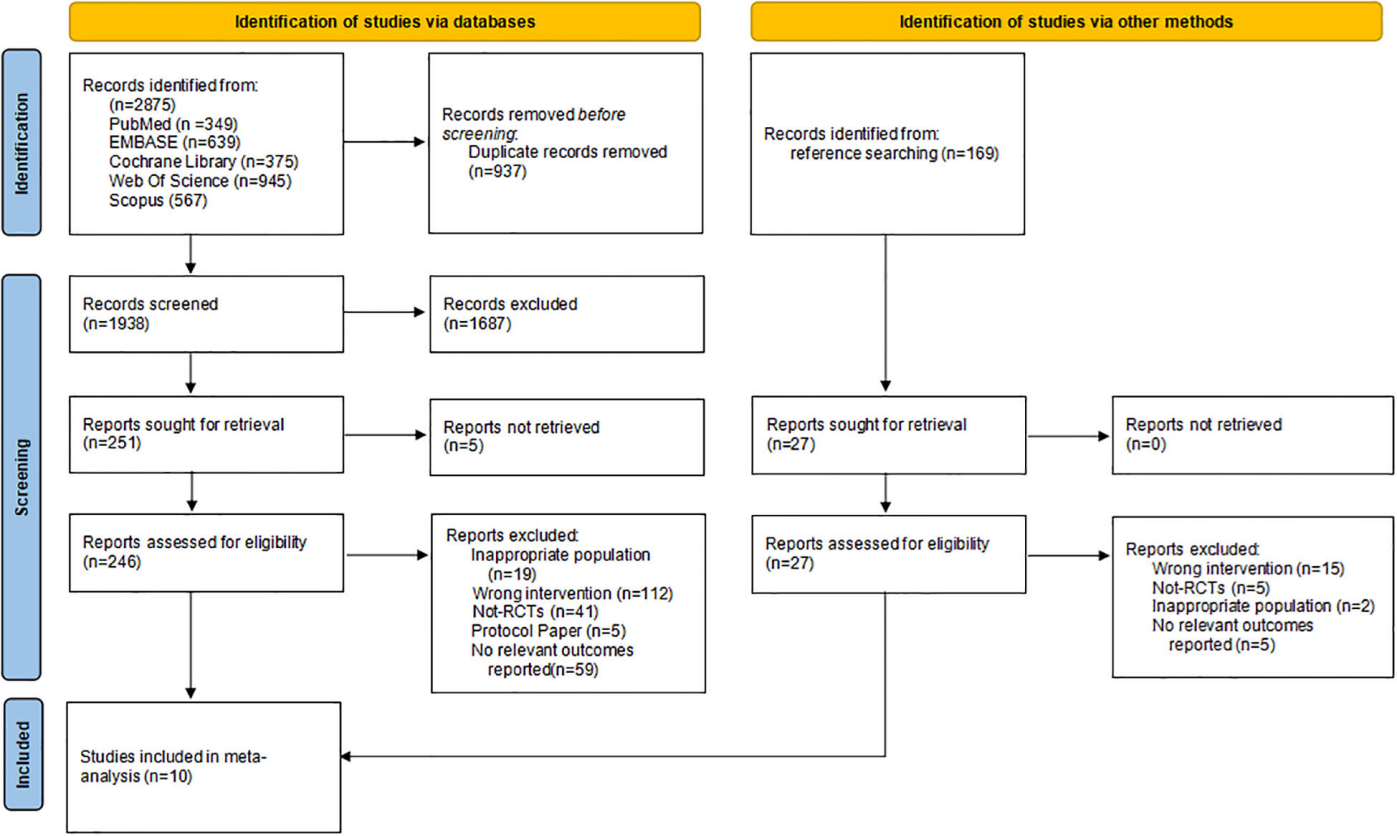
PRISMA flowchart describing study identification and selection process.

The review process is illustrated in the PRISMA flow diagram (**Figure 1**). The search strategy identified 2875 unique articles for title and abstract screening. After screening, 251 full-text articles were retrieved, of which 236 articles were excluded for evaluation. Among the excluded articles, 112 were excluded for inappropriate interventions, 41 for study design, 19 for inappropriate population, 5 for protocol papers, and 59 for no relevant outcomes. A manual search from other sources (e.g., backward and forward citation searches) identified 169 records, but identified no further included articles. Finally, 10 articles were considered eligible for inclusion.

### Study characteristics

Ten randomized controlled trials encompassing 930 women with polycystic ovary syndrome (mean age range 24.7–31.8 years) were included (**Table 1**). Studies were published from 2007 to 2024, with eight of ten (80%) appearing between 2021 and 2024. Trials were conducted in China (n = 6) (21, 24–28), Iran (n = 1) (22), South Korea (n = 1) (23), Norway (n = 1) (29), and Italy (n = 1) (30). Digital health interventions fell into five main categories: WeChat−based multidisciplinary management (n = 4) (21, 24, 25, 27), App−based mHealth (n = 2) (23, 26), motivational messaging via WhatsApp (n = 1) (22), video−assisted education (n = 1) (28), and device−linked self−monitoring (n = 2) (29, 30). Intervention durations ranged from a single session to 24 weeks (most 8–12 weeks). Only two studies (22, 25) reported health behavior-related measures using the Health−Promoting Lifestyle Profile II (HPLP−II).

**Table 1:** Characteristics of the included studies.

Author(year), Country	Sample characteristics N; Age		Description of digital health interventions	Description of control	Outcomes measures
	Intervention group	Control group			
Dilimulati (2024), China (21)	40; 27.60 (1.37)	40; 27.95 (1.38)	WeChat-based digital intervention (3 modules for lifestyle: diet, exercise, sleep) for 12 weeks	Metformin 1000mg/day for 12 weeks	BMI Weight Waist Circumference
Hamzehgardeshi (2024), Iran (22)	30; 30.97 (4.18)	30; 28.67 (5.30)	Five weekly group motivational interview sessions via WhatsApp (audio/video/written, PowerPoint), follow-up support via WhatsApp	Routine infertility and PCOS care	HPLP-II Score
Lee (2023), South Korea (23)	14; 28.50 (4.83)	14; 25.36 (4.40)	12-week lifestyle modification program using a mobile app, includes daily tracking of diet, exercise, and symptoms with weekly feedback	Usual care with evidence-based PCOS leaflet	Weight
Ou (2023), China (24)	50; 27.54 (3.06)	50; 26.91 (3.08)	“Internet +” multidisciplinary management model oriented by nurse specialists via the WeChat platform	Traditional treatment and routine nursing	BMI Waist Circumference Anxiety Depression
Guo (2022), China (25)	40; 24.95 (4.02)	40; 25.98 (4.05)	WeChat-based multidimensional life management intervention (diet/exercise/education) for 6 months	Routine care (unstructured patient education) for 6 months	BMI Waist Circumference HPLP-II Score
Wang (2022), China (26)	51; 24.72 (4.20)	49; 24.94 (4.31)	TTM-based mobile health application (Home of PCOS), includes modules for behavioral stage assessment, self-monitoring, peer support, and expert consultation.	Routine care: advice on maintaining physical activity and a balanced diet from gynecologists	BMI Waist Circumference Anxiety Depression
Sang (2022), China (27)	39; 28.9 (3.1)	40; 30.1 (4.5)	Lifestyle modification via WeChat app (diet, exercise, education, support) 3 months	Routine care: drug and lifestyle education (diet, exercise, weight recording) 3 months	BMI
Liu (2021), China (28)	164; 31.79 (3.38)	132; 32.31 (3.79)	individual sessions of face-to-face instruction and video teaching for 1-h duration	Guidance on diet, exercise, and weight gain	Weight
Almenning (2015), Norway (29)	8; 27.2 (5.5)	9; 27.2 (5.5)	Heart rate monitors are linked to an online platform to track exercise intensity and compliance, with weekly data reviews ensuring accuracy	Usual care	BMI Weight Waist Circumference
Vigorito (2007), Italy (30)	45; 21.7 (2.3)	45; 21.9 (1.9)	Exercise with continuous electrocardiographic monitoring	Usual care	BMI Waist Circumference

NA, not available.

BMI, body mass index; HPLP-II, Health-Promoting Lifestyle Profile II; PCOS, Polycystic Ovary Syndrome.

### Risk-of-bias assessment

**Figure 2:**
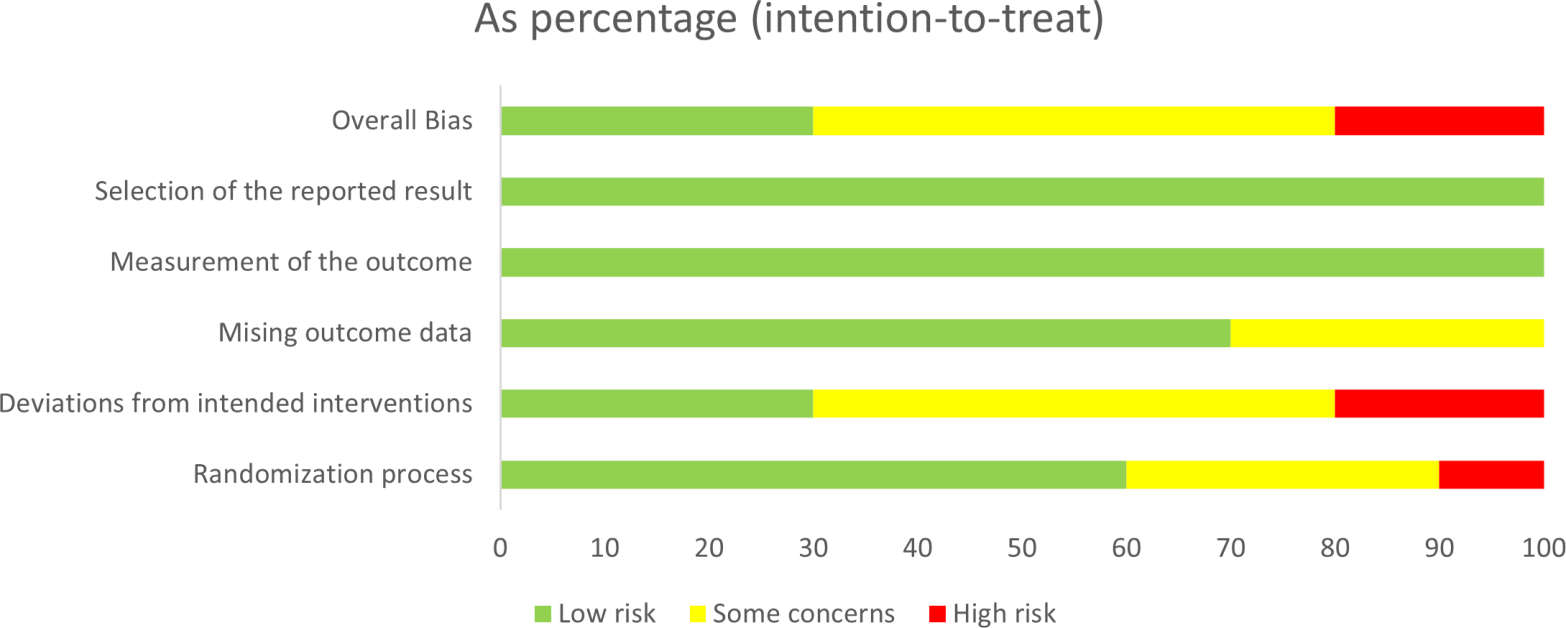
Risk of bias summary for all the included studies.

**Figure 3:**
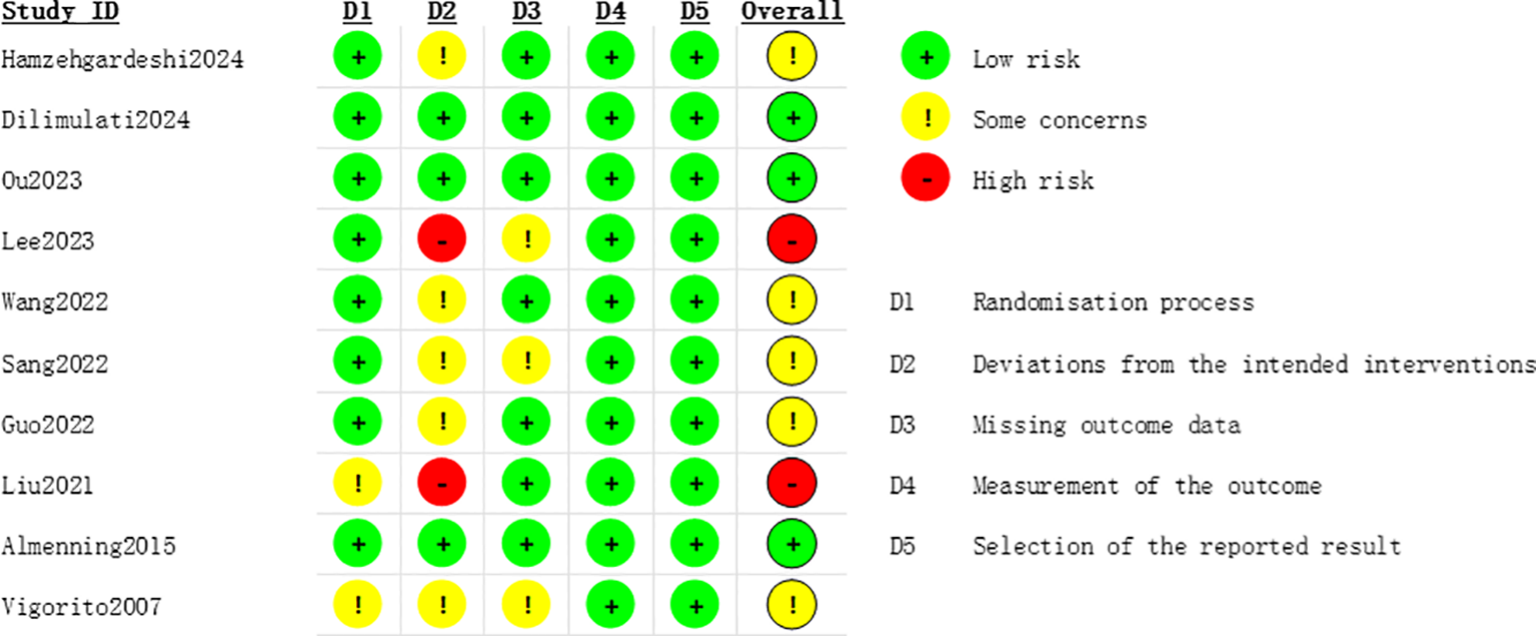
Summary of distribution of different biases.

Details of the risk of bias assessment in individual trials are provided in **Figures 2**, **3**. Among the 10 included trials, five (50%) trials had some concerns of bias, three (30%) trials had low risk of bias (21, 24, 29), and two (20%) trials had a high risk of bias (23, 28). The studies by Liu et al. (28) and Lee et al. (23) were graded at high risk of bias because of deviations from the intended interventions.

### Effects of interventions

The GRADE evidence profiles summarizing the comparative effectiveness of digital health interventions versus non-digital controls for short-term and medium-term outcomes are presented in **Tables 2**, **3**, respectively.

**Table 2:** The GRADE summary of findings for short-term outcomes.

Digital compared to non-digital for polycystic ovary syndrome					
Outcomes	Anticipated absolute effects^*^ (95% CI)		Relative effect (95% CI)	№ of participants (studies)	Certainty of the evidence (GRADE)
	Risk with non-digital	Risk with digital			
BMI	–	MD **1.19 lower** (1.84 lower to 0.55 lower)	–	340 (5 RCTs)	⊕⊕⊕◯ Moderate^a^
Weight	–	SMD **0.04 lower** (0.23 lower to 0.15 higher)	–	421 (4 RCTs)	⊕⊕⊕⊕ High
Waist Circumference	–	MD **2.14 lower** (3.11 lower to 1.17 lower)	–	361 (5 RCTs)	⊕⊕⊕◯ Moderate^a^
HPLP-II Total Score	–	SMD **1.61 higher** (1.2 higher to 2.01 higher)	–	126 (2 RCTs)	⊕⊕⊕◯ Moderate^a^
HPLP-II Total Score Physical Activity	–	SMD **1.43 higher** (1.04 higher to 1.83 higher)	–	126 (2 RCTs)	⊕⊕⊕◯ Moderate^a^
HPLP-II Total Score Nutrition	–	SMD **0.83 higher** (0.11 lower to 1.78 higher)	–	126 (2 RCTs)	⊕⊕◯◯ Low^a,b^
HPLP-II Health Responsibility	–	SMD **1.02 higher** (0.64 higher to 1.39 higher)	–	126 (2 RCTs)	⊕⊕⊕◯ Moderate^a^
HPLP-II Total Score Interpersonal Relationships	–	SMD **0.96 higher** (0.59 higher to 1.33 higher)	–	126 (2 RCTs)	⊕⊕⊕◯ Moderate^a^
HPLP-II Total Score Spiritual Growth	–	SMD **1.25 higher** (0.87 higher to 1.64 higher)	–	126 (2 RCTs)	⊕⊕⊕◯ Moderate^a^
HPLP-II Total Score Stress Management	–	SMD **1.17 higher** (0.79 higher to 1.55 higher)	–	126 (2 RCTs)	⊕⊕⊕◯ Moderate^a^

*The risk in the intervention group (and its 95% confidence interval) is based on the assumed risk in the comparison group and the relative effect of the intervention (and its 95% CI).

CI, confidence interval; MD, mean difference; OR, odds ratio; SMD, standardized mean difference.

GRADE Working Group grades of evidence.

High certainty: we are very confident that the true effect lies close to that of the estimate of the effect.

Moderate certainty: we are moderately confident in the effect estimate: the true effect is likely to be close to the estimate of the effect, but there is a possibility that it is substantially different.

Low certainty: our confidence in the effect estimate is limited: the true effect may be substantially different from the estimate of the effect.

Very low certainty: we have very little confidence in the effect estimate: the true effect is likely to be substantially different from the estimate of effect.

Explanations

^a^Total participants in the meta-analysis ≤ 400.

^b^I² > 75% (serious heterogeneity).

Bold values indicate effects favoring the intervention group.

**Table 3:** The GRADE summary of findings for middle-term outcomes.

Digital compared to non-digital for polycystic ovary syndrome					
Outcomes	Anticipated absolute effects^*^ (95% CI)		Relative effect (95% CI)	№ of participants (studies)	Certainty of the evidence (GRADE)
	Risk with non-digital	Risk with digital			
BMI		MD **2.46 lower** (3.04 lower to 1.88 lower)	–	274 (3 RCTs)	⊕⊕⊕◯ Moderate^a^
Waist Circumference		MD **4.65 lower** (6.7 lower to 2.6 lower)	–	174 (2 RCTs)	⊕⊕⊕◯ Moderate^a^
Depression	–	SMD **0.85 lower** (1.17 lower to 0.53 lower)	–	200 (2 RCTs)	⊕⊕⊕◯ Moderate^a^
Anxiety	–	SMD **0.95 lower** (1.33 lower to 0.56 lower)	–	200 (2 RCTs)	⊕⊕⊕◯ Moderate^a^

*The risk in the intervention group (and its 95% confidence interval) is based on the assumed risk in the comparison group and the relative effect of the intervention (and its 95% CI).

CI, confidence interval; MD, mean difference; OR, odds ratio; SMD, standardized mean difference.

GRADE Working Group grades of evidence.

High certainty: we are very confident that the true effect lies close to that of the estimate of the effect.

Moderate certainty: we are moderately confident in the effect estimate: the true effect is likely to be close to the estimate of the effect, but there is a possibility that it is substantially different.

Low certainty: our confidence in the effect estimate is limited: the true effect may be substantially different from the estimate of the effect.

Very low certainty: we have very little confidence in the effect estimate: the true effect is likely to be substantially different from the estimate of effect.

Explanations

^a^Total participants in the meta-analysis ≤ 400.

Bold values indicate effects favoring the intervention group.

### Short-term outcomes

**Figure 4:**
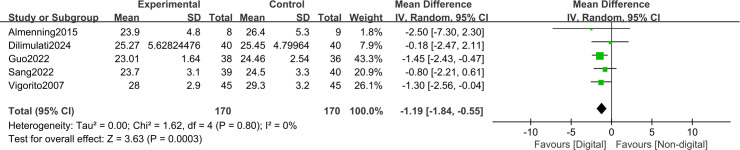
Forest plot for the short-term BMI.

**Figure 5:**
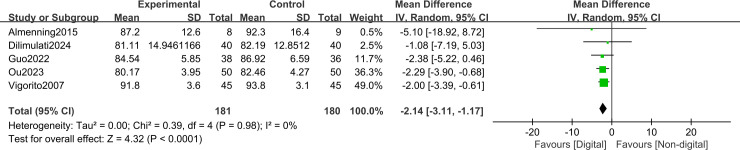
Forest plot for the short-term waist circumference.

**Figure 6:**
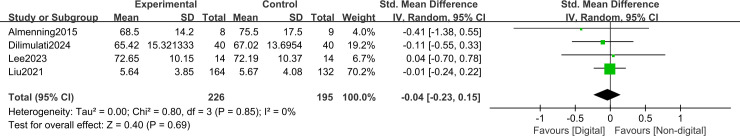
Forest plot for the short-term weight.

**Figure 7:**
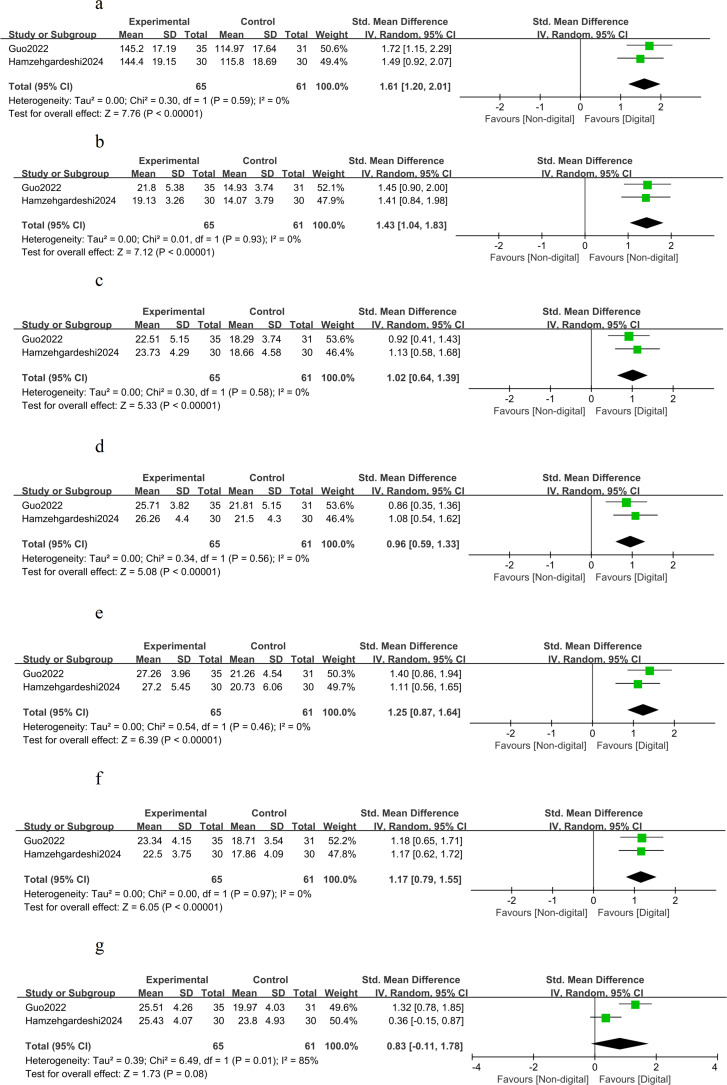
Forest plots for the short-term HPLP-II: **(a)** total HPLP-II score; **(b)** physical activity subscale; **(c)** health responsibility subscale; **(d)** interpersonal relationships subscale; **(e)** spiritual growth subscale; **(f)** stress management subscale; and **(g)** nutrition subscale.

In the short term (≤3 months), DHIs were associated with significant reductions in key anthropometric measures compared with non-DHIs. A pooled meta-analysis of five trials (n = 340) demonstrated a reduction in BMI of −1.19 kg/m² (MD, -1.19; 95% CI, −1.84 to −0.55; I² = 0%; GRADE: moderate) (**Figure 4**). Meanwhile, a meta-analysis of five trials (n=361) showed a reduction in waist circumference of −2.14 cm (MD, -2.14; 95% CI, −3.11 to −1.17; I² = 0%; GRADE: moderate) (**Figure 5**). Across four studies (n = 421), there was no significant effect on body weight (SMD, −0.04; 95% CI, −0.23 to 0.15; I² = 0%; GRADE: high) (**Figure 6**). In addition, health behaviors measured by HPLP−II (2 studies; n = 126) showed large improvements compared with non-DHIs: total HPLP−II score (SMD, 1.61; 95% CI, 1.20 to 2.01; I² = 0%; GRADE: moderate) (**Figure 7a**), physical activity subscale (SMD, 1.43; 95% CI, 1.04 to 1.83; I² = 0%; GRADE: moderate) (**Figure 7b**), health responsibility subscale (SMD, 1.02; 95% CI, 0.64 to 1.39; I² = 0%; GRADE: moderate) (**Figure 7c**), interpersonal relationships subscale (SMD, 0.96; 95% CI, 0.59 to 1.33; I² = 0%; GRADE: moderate) (**Figure 7d**), spiritual growth subscale (SMD, 1.25; 95% CI, 0.87 to 1.64; I² = 0%; GRADE: moderate) (**Figure 7e**), and stress management subscale (SMD, 1.17; 95% CI, 0.79 to 1.55; I² = 0%; GRADE: moderate) (**Figure 7f**). The HPLP−II nutrition subscale, however, did not reach statistical significance (SMD, 0.83; 95% CI, −0.11 to 1.78; I² = 84%; GRADE: low) compared with non-DHIs (**Figure 7g**).

### Medium-term outcomes

**Figure 8:**
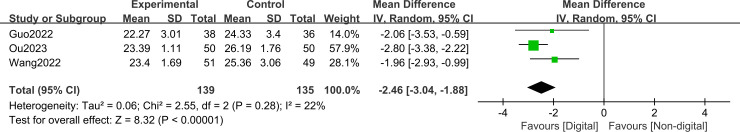
Forest plot for the medium-term BMI.

**Figure 9:**
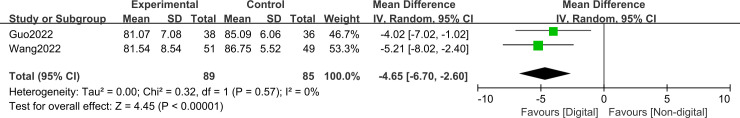
Forest plot for the medium-term waist circumference.

**Figure 10:**
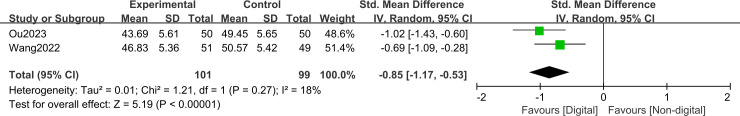
Forest plot for the medium-term depression.

**Figure 11:**
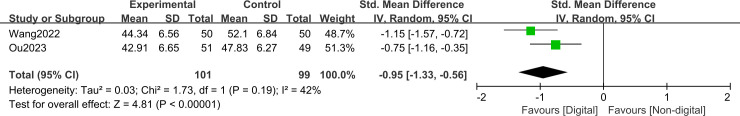
Forest plot for the medium-term anxiety.

In the middle term (3–6 months), a pooled meta-analysis of three trials (n = 274) reported a BMI reduction of −2.46 kg/m² (95% CI, −3.04 to −1.88; I² = 22%; GRADE: moderate) **(Figure 8**), and two trials (n = 174) demonstrated a moderate decrease in waist circumference (MD, −4.65; 95% CI, −6.70 to −2.60; I² = 0%; GRADE: moderate) compared with non-DHIs (**Figure 9**). Moreover, DHIs also yielded significant psychological improvements compared with non-DHIs: depressive symptoms were reduced (2 studies; n = 200; SMD, −0.85; 95% CI, −1.17 to −0.53; I² = 17%; GRADE: moderate) (**Figure 10**) as were anxiety levels (SMD, −0.95; 95% CI, −1.33 to −0.56; I² = 42%; GRADE: moderate) (**Figure 11**).

## Discussion

To our knowledge, this is the first meta-analysis to show that digital health interventions yielded modest, but significant improvements in anthropometric and healthy behavior measures among women with PCOS. Specifically, based on moderate-certainty evidence, digital health interventions produced significant reductions in BMI and waist circumference in both short‐term (≤3 months) and medium‐term (3–6 months) follow-up. These changes are comparable to weight‐related benefits seen with structured weight‐loss programs in PCOS, which are known to improve metabolic features of the syndrome (26, 31). By contrast, based on high-certainty evidence, there was no significant effect on absolute body weight in the short term. The significant BMI reduction despite non-significant changes in absolute body weight can be partly attributed to metric sensitivity differences. BMI incorporates height squared in its denominator, amplifying small weight losses into more pronounced BMI shifts, whereas absolute weight change may require larger mass reductions to reach statistical significance (32).

Notably, we observed substantial gains in health behaviors. Based on moderate-certainty evidence, digital interventions markedly improved multiple domains of the HPLP-II, such as physical activity, health responsibility, interpersonal relationships, spiritual growth, and stress management, indicating enhanced adoption of healthy behaviors. The only exception in our health behavior outcomes was the nutrition subscale of HPLP-II, which showed no statistically significant change; this analysis exhibited high heterogeneity (I^2^ = 84%) and very low-certainty evidence, so the effect on dietary behaviors remains uncertain. The heterogeneity observed in the meta-analysis results of the nutrition-related outcomes may stem from inconsistencies in intervention characteristics. Specifically, Guo et al.’s study (25) employed a comprehensive intervention lasting six months, whereas Hamzehgardeshi’s study (22) utilized a single online Five weekly group motivational interview lasting only two months. Especially, our finding of no change in dietary-behavior scores contrasts with Wang et al.’s report of improved diet adherence (26), possibly reflecting differences in dietary metrics or the limited number of studies in our meta-analysis. Improvements in psychosocial outcomes were also robust: based on moderate-certainty evidence, depressive and anxiety symptoms were significantly reduced in medium‐term follow-up. This finding aligns with evidence that digital therapies can effectively alleviate depression and anxiety symptoms (33).

## Limitations

There are several limitations to acknowledge. First, many pooled outcomes are based on a small number of trials with modest sample sizes (often only 2–5 studies), limiting precision. In particular, the nutrition subscale was estimated from just two studies, which explained the very high statistical heterogeneity (I^2^ = 84%) and low certainty using the GRADE approach; thus, this result should be interpreted with caution. Second, the trials generally had short follow-up (≤6 months), so long-term sustainability of benefits is unknown. Indeed, previous systematic reviews have noted moderate-to-severe risk of bias and poor retention in many web-based health programs (33). Third, the heterogeneity across interventions (different technologies, content, and intensities) and comparators introduces variability in effect estimates. Finally, as with any meta-analysis, unmeasured publication bias or selective reporting cannot be excluded.

## Future considerations

Future research should aim to bolster and broaden this study’s evidence. Large-scale, robust RCTs with extended durations are necessary to confirm short-to-medium term effects and assess whether long-term health benefits are also sustained. Studies should standardize outcomes, particularly for diet and psychosocial factors, and report attrition/engagement data to mitigate bias. Enhancing user engagement is crucial, potentially through interactive coaching, gamification, or social support. Tailored, theory-driven interventions, like the Transtheoretical Model app, may boost adherence and effectiveness (34). Future efforts should focus on dietary changes using objective metrics and explore combining digital tools with in-person support, considering diverse PCOS populations, including lean patients and various age groups. Finally, implementation studies are needed to translate these findings into practice – for example, assessing how to integrate mobile lifestyle programs into routine PCOS care pathways. Incorporating these scalable, cost-effective, and customizable digital health interventions into clinical management pathways for women with PCOS—via a streamlined referral and onboarding process, designated clinician or care-coordinator oversight, and routine monitoring of engagement and key outcomes—can facilitate the delivery of comprehensive, patient-centered health interventions.

## Conclusion

In summary, our systematic review and meta-analysis indicate that digital health interventions can produce significant improvements in BMI, waist circumference, and various health behaviors, along with meaningful reductions in depression and anxiety for women with PCOS. While the evidence is promising, especially for short-term and medium-term outcomes, it is tempered by limitations in study design and evidence quality. Therefore, digital lifestyle therapies should be considered a useful component of PCOS care, but further high-quality research is needed to confirm long-term efficacy and optimize intervention design and delivery.

## Data Availability

The original contributions presented in the study are included in the article/**Supplementary Material**. Further inquiries can be directed to the corresponding authors.
