# Supplementary material for: The effectiveness of digital health interventions on anthropometric and healthy behavior in women with polycystic ovarian syndrome: a systematic review with meta-analysis
Source: Front Endocrinol (Lausanne). 2026 Feb 27;17:1676915. doi: 10.3389/fendo.2026.1676915 (PMC12982043; doi:10.3389/fendo.2026.1676915)
Supplement: Supplementary file 1 [file Table1.docx]

**Table A. Example search strategy.**

The search strategy for PubMed was shown below. It was adapted for the other databases.

| **Pubmed**  ("Polycystic Ovary Syndrome"[MeSH Terms] OR ("Polycystic Ovar*"[Title/Abstract] OR "PCOS"[Title/Abstract] OR "PCOD"[Title/Abstract] OR "Sclerocystic Ovar*"[Title/Abstract] OR "Stein Leventhal"[Title/Abstract])) AND ((digital[Title/Abstract] OR "electronic intervention*"[Title/Abstract] OR "internet"[Title/Abstract] OR "mobile phone"[Title/Abstract] OR "smartphone"[Title/Abstract] OR "app"[Title/Abstract] OR "E-health"[Title/Abstract] OR "online"[Title/Abstract] OR "text message"[Title/Abstract] OR "multimedia message"[Title/Abstract] OR "mobile application"[Title/Abstract] OR "social media"[Title/Abstract] OR "email"[Title/Abstract] OR "cell phone"[Title/Abstract] OR "mhealth"[Title/Abstract] OR "m-health"[Title/Abstract] OR "telehealth"[Title/Abstract] OR "twitter"[Title/Abstract] OR "facebook"[Title/Abstract] OR "iphone"[Title/Abstract] OR "ipad"[Title/Abstract] OR "webinar"[Title/Abstract] OR "electronic aids"[Title/Abstract] OR "ehealth"[Title/Abstract] OR "web"[Title/Abstract] OR "website"[Title/Abstract] OR "computer"[Title/Abstract] OR "sms"[Title/Abstract] OR "msm"[Title/Abstract] OR "android"[Title/Abstract])) AND ("randomized controlled trial"[Publication Type] OR "random*"[Title/Abstract] OR "placebo"[Title/Abstract]) | **Adapted for:**  Cochrane, Embase,  Web of Science, and Scopus. |
| --- | --- |

**Table B. Detailed GRADE rating criteria：**

1. **Risk of bias**: If half of the study participants derive from trials judged at high risk of bias, we downgrade the certainty by one level.
2. **Inconsistency**:
   - I² > 75% (serious heterogeneity): downgrade by one level
3. **Imprecision**:
   - Total participants in the meta-analysis ≤ 400: downgrade by one level
4. **Publication bias**: Detection of bias via statistical tests leads to a one-level downgrade. If there were not able to examine publication bias, we did not downgrade for publication bias.
5. **Indirectness**: When over 50% of participants, interventions, comparators, or outcomes do not directly match the review’s eligibility criteria, we lower the certainty by one level.
